# The vestibular system: a spatial reference for bodily self-consciousness

**DOI:** 10.3389/fnint.2014.00031

**Published:** 2014-04-17

**Authors:** Christian Pfeiffer, Andrea Serino, Olaf Blanke

**Affiliations:** ^1^Center for Neuroprosthetics, School of Life Sciences, Ecole Polytechnique Fédérale de LausanneLausanne, Switzerland; ^2^Laboratory of Cognitive Neuroscience, Brain Mind Institute, School of Life Sciences, Ecole Polytechnique Fédérale de LausanneLausanne, Switzerland; ^3^Department of Psychology, Alma Mater Studiorum, University of BolognaBologna, Italy; ^4^Department of Neurology, University Hospital GenevaGeneva, Switzerland

**Keywords:** bodily self-consciousness, multisensory integration, first-person perspective, self-location, self-motion, mental spatial transformation, body representation, vestibular cortex

## Abstract

Self-consciousness is the remarkable human experience of being a subject: the “I”. Self-consciousness is typically bound to a body, and particularly to the spatial dimensions of the body, as well as to its location and displacement in the gravitational field. Because the vestibular system encodes head position and movement in three-dimensional space, vestibular cortical processing likely contributes to spatial aspects of bodily self-consciousness. We review here recent data showing vestibular effects on first-person perspective (the feeling from where “I” experience the world) and self-location (the feeling where “I” am located in space). We compare these findings to data showing vestibular effects on mental spatial transformation, self-motion perception, and body representation showing vestibular contributions to various spatial representations of the body with respect to the external world. Finally, we discuss the role for four posterior brain regions that process vestibular and other multisensory signals to encode spatial aspects of bodily self-consciousness: temporoparietal junction, parietoinsular vestibular cortex, ventral intraparietal region, and medial superior temporal region. We propose that vestibular processing in these cortical regions is critical in linking multisensory signals from the body (personal and peripersonal space) with external (extrapersonal) space. Therefore, the vestibular system plays a critical role for neural representations of spatial aspects of bodily self-consciousness.

## Introduction

Humans’ experience as subject (“I”, the self) is typically bound to the spatial dimensions of the physical body. This is expressed by the concept of bodily self-consciousness, which consists of several aspects including the experience that “I” am localized at a specific place and spatial volume (self-location), the experience that “I” take an experiential and visuospatial perspective of the world (first-person perspective), the experience that “I” identify with the body as a whole (self-identification) as opposed to feeling ownership for a body part, and that “I” am causing actions through the body (sense of agency) (Haggard et al., 2003; Jeannerod, 2003; Blanke and Metzinger, 2009; Blanke, 2012; Metzinger, 2013; Serino et al., 2013). This review will mainly focus on what we call spatial aspects of bodily self-consciousness, i.e., self-location and first-person perspective. These phenomenal experiences are defined by spatial parameters, such as the location and volumetric expansion of the self and the origin and direction of perspective (Blanke and Metzinger, 2009). In contrast, we will be less concerned with non-spatial aspects of bodily self-consciousness, i.e., self-identification and agency. These phenomenal experiences are invariant to changes in spatial parameters (see Metzinger, 2013 for a discussion on self-identification without a body in lucid dreams and during out-of-body experiences).

Experimental research shows that both spatial and non-spatial aspects of bodily self-consciousness emerge from pre-reflective and non-conceptual representations of bodily signals in the brain (Metzinger, 2003; Gallagher, 2005; Blanke and Metzinger, 2009; Ehrsson, 2012). Those are sensory signals from exteroception, such as visual and auditory signals (e.g., Ehrsson, 2007; Lenggenhager et al., 2007; Tajadura-Jiménez et al., 2009), from somatosensation, such as tactile and proprioceptive signals (e.g., Seizova-Cajic et al., 2007; Palluel et al., 2011; for reviews see Haggard et al., 2003; Serino and Haggard, 2010) and from interoception, such as cardiac, nociceptive, and thermal signals (Hänsel et al., 2011; Aspell et al., 2013; for an interoception-based account on consciousness see Craig, 2002, 2009). Altogether, these experimental studies imply that by integrating multisensory signals the brain generates a coherent spatial representation of body parts, the body as a whole, and the body as related to the external world.

However, much less is known about the role of the vestibular system for bodily self-consciousness. Because the vestibular system encodes the position and movement of the head in three-dimensional space, and because in the central nervous system vestibular signals are strongly integrated with motor, visual, somatosensory and proprioceptive signals (Grüsser et al., 1990a,b; Gu et al., 2007; Prsa et al., 2012), central vestibular processing may be an important contributor to the neural computations underlying spatial aspects of bodily self-consciousness. Specifically, vestibular signals might contribute in generating a spatial representation of the body as a whole with respect to the external world, i.e., in the gravitational field in particular. These vestibular signals might be critical for updating whole body representation while this one moves in external space. Accordingly, the vestibular system would encode spatial references for self-location and first-person perspective.

This review summarizes and critically discusses both direct and indirect evidence for this proposal. While topics in the fields of bodily self-consciousness and central vestibular processing have been mostly studied in isolation, with this review article we hope to motivate a converging approach from these exciting research fields.

The review is divided in three parts. In the first part, we briefly introduce the vestibular system and then summarize current knowledge about the role of vestibular processing for spatial aspects of bodily self-consciousness. We conclude the first part by several questions that remain open to experimental research. In the second part, we review experimental data about vestibular contributions to cognitive and perceptual processes that involve spatial representations of the bodily self with respect to the external world. We think that these self-related processes draw on similar functional mechanisms as spatial aspects of bodily self-consciousness, and we discuss these experimental data as indirect evidence for vestibular contributions to spatial aspects of bodily self-consciousness. The third and final part of this review is concerned with the neural correlates of vestibular processing underlying self-location and first-person perspective. We propose that self-location and first-person perspective are encoded by a posterior cortical network consisting of the temporoparietal junction (TPJ), i.e., a region that has been causally linked to bodily self-consciousness, and three vestibular cortex regions, i.e., the parietoinsular vestibular cortex (PIVC), the medial superior temporal region (MST), and the ventral intraparietal region (VIP), which together perform the necessary computation subserving a multisensory spatial reference for bodily self-consciousness. We discuss the known functional properties of these regions and their putative role in bodily self-consciousness. Together we provide an argument supporting our hypothesis and present a testable outlook for future research for the study of vestibular processing in spatial aspects of bodily self-consciousness.

## Part one: the vestibular system and bodily self-consciousness: current knowledge and open questions

### The vestibular system

The vestibular system encodes linear and rotatory acceleration of the head. It senses constant linear acceleration by earth gravity and thus signals to the brain head movement and position with respect to a constant gravitational acceleration. The vestibular system contributes to a variety of central nervous system functions including motor control, e.g., stabilizing gaze by the vestibular-ocular reflex (Schwarz, 1976), body posture (Pozzo et al., 1990), perception, e.g., of verticality (Lopez et al., 2007), and of self-motion (Brandt et al., 1998). Moreover, it also contributes to cognition, e.g., spatial navigation and memory (Arthur et al., 2009), and bodily self-consciousness (Blanke et al., 2002; Pfeiffer et al., 2013), which is the main topic of this review.

#### Peripheral system

**Figure 1:**
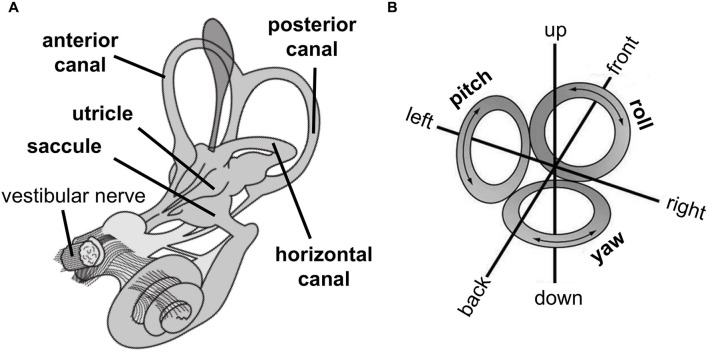
**(A)** Peripheral vestibular organs in the inner ear consist of otoliths, i.e., utricle and saccule, which sense linear acceleration, and semicircular canals, i.e., anterior, posterior, and horizontal canal, which sense rotational acceleration. The vestibular nerve projects signals from otoliths and semicircular canals to the central nervous system. **(B)** The vestibular system encodes movement in three-dimensional space denoted as linear movements, i.e., in front, back, left, right, up, and down direction (by otolith organs) and rotational movements, i.e., yaw (by the horizontal canal) and pitch and roll (by both anterior and posterior canal). (Images are derivatives of works by NASA, licensed under creative commons.)

The peripheral vestibular organs are located bilaterally in the head and are part of the inner ear (Figure 1A). They consist of two otolith organs (utricle and saccule) that sense linear acceleration, e.g., by head motion or gravitational force, and three semicircular canals (anterior, posterior and horizontal canal) that sense rotational acceleration around three cardinal axes (yaw, roll, pitch, Figure 1B). Thus, the vestibular sensory organs encode head position and movement in three-dimensional space.

Experimental research on the vestibular system has mainly used two approaches in order to stimulate the vestibular system, i.e., by natural and artificial stimulation. Natural vestibular stimulation can be experimentally induced by head accelerations, e.g., by passive whole-body rotation or translation (e.g., Prsa et al., 2012; van Elk and Blanke, 2014) that are sensed by the semicircular canals or otolith organs respectively. Natural vestibular stimulation may be given under terrestrial conditions by constant gravitational forces due to the attraction exerted by the earth on mass. Because the otolith organs sense constantly the vector of constant acceleration by gravity, static body or head tilts with respect to gravity can be used to naturally stimulate the otolith organs. The effects of weightlessness on vestibular processing have been studied in spacecrafts in orbit or in aircrafts during prolonged free fall (i.e., up to several months duration) or during parabolic flight (i.e., less than a minute duration).

Artificial peripheral vestibular stimulation techniques are: monopolar or bipolar electrical stimulation at the mastoids (Galvanic Vestibular Stimulation, GVS), thermal irrigation of one or both ear canals (Caloric Vestibular Stimulation, CVS), and auditory stimulation on headphones (clicks and short-tone bursts). These stimulation techniques activate the semicircular canals, otolith organs, the vestibular nerve, or a combination of the previous. Notably, these artificial stimulations co-activate nociceptive, thermal, and auditory sensory receptors—for comparison of these techniques and cortical processing see Lopez et al. (2012b).

#### Vestibular cortex

The central nervous system vestibular pathway consists of: (i) vestibular nerve projections from the vestibular organs to the vestibular nucleus in the brainstem; (ii) projections from the brainstem to thalamic nuclei, cerebellum, and spinal cord; and (iii) projections from the thalamus to the cerebral cortex. The interested reader can find comprehensive reviews on the peripheral and central vestibular system in Goldberg et al. (2012) and Lopez and Blanke (2011).

While for vision, audition, and somatosensation specific unisensory primary cortices have been identified, no such unisensory vestibular cortex seems to exist in the human brain. Rather, vestibular cortex is considered as any cortical region receiving vestibular input from the thalamus and is a distributed cortical network that overlaps with multisensory and motor representations from vision, somatosensation, proprioception, and action (Lopez and Blanke, 2011).

Electrophysiological recordings in non-human primates have identified vestibular inputs in several cortical regions including the somatosensory cortex (area 3aN, area 3aH, area 2v), PIVC, dorsal MST, medial temporal cortex, frontal cortex (frontal eye field and supplementary eye field), and cingulate cortex (Grüsser et al., 1990b; Guldin et al., 1992; Bremmer et al., 2002; Gu et al., 2007). These recordings revealed therefore thalamocortical projections to all major cortical lobes except the occipital lobe.

In order to measure human vestibular cortical processing, many studies have used functional magnetic resonance imaging (fMRI). While fMRI has the advantage of high spatial resolution and non-invasiveness, it is worth noting that studying vestibular processing in fMRI has several limitations. First, participants are required to lie supine and must avoid head movements, which differs from conditions of vestibular stimulation in natural context, typically involving different head postures and movements. Secondly, in order to stimulate the peripheral vestibular organs artificial stimulation techniques (GVS, CVS, clicks) are used. These co-activate other sensory modalities and complicate the interpretation of observed brain activation as purely vestibular Lopez et al. (2012b). Finally, the static magnetic field of the MR scanner induces a constant vestibular stimulus that, depending on participant’s head position, differently activates the vestibular sensory organs and can even induce vertigo (Mian et al., 2013). Thus, there are limitations with current fMRI approaches to study central vestibular processing. It will be an important future goal to develop novel approaches allowing more natural and specific vestibular stimulation during non-invasive neuroimaging in humans.

### Vestibular contributions to bodily self-consciousness

#### Theory

It has been proposed that bodily self-consciousness is based on the brain’s multisensory integration of visual, vestibular, somatosensory, proprioceptive and motor signals (Haggard et al., 2003; Blanke et al., 2004; Blanke and Mohr, 2005). This theory distinguishes between personal (including also peripersonal) space, which is a volume of space occupied by the physical body and the space immediately surrounding the body, and extrapersonal space, that is the space outside of personal space. The theory proposes that the vestibular system is critically involved in integrating sensory signals from personal space (e.g., somatosensory, proprioceptive, visual, and auditory signals) with sensory signals from extrapersonal space (e.g., visual and auditory signals). It was proposed that particularly otolithic vestibular signals about constant gravitational acceleration provide a world-centered reference for the bodily self. By means of multisensory integration between personal and extrapersonal space the brain generates a spatial representation of the body as a whole, with a given location and orientation with respect to the external world, i.e., bodily self-consciousness. In line with this theory, Lopez et al. (2008) argued that vestibular otolithic signals are highly relevant for spatial aspects of bodily self-consciousness, i.e., self-location and first-person perspective, which depend on signals from both personal and extrapersonal space, and that vestibular signals may be less relevant for non-spatial aspects of bodily self-consciousness, e.g., self-identification, which depend mainly on signals from personal space and relate mostly to the body itself, rather than to the body relative to the external world (see also Blanke, 2012).

#### Clinical data

The strongest support for the proposal that vestibular processing contributes to bodily self-consciousness comes from observations in neurological patients suffering from out-of-body experience who show a three-way disembodiment of their bodily self-consciousness (Devinsky et al., 1989; Blanke et al., 2002, 2004; Brandt et al., 2005; De Ridder et al., 2007; Ionta et al., 2011; Pfeiffer et al., 2013). During an out-of-body experience patients typically identify with an illusory body in external space (disembodied self-identification), feel to be elevated above their physical body (disembodied self-location), and to have an elevated visuospatial perspective directed back downward to the physical body (disembodied first-person perspective).

Out-of-body experience in some neurological patients were caused by damage (Ionta et al., 2011), dysfunction (Blanke et al., 2004), or electrical stimulation (Blanke et al., 2002) at the TPJ, i.e., a brain region that receives strong vestibular inputs (Lopez et al., 2008, 2012b; zu Eulenburg et al., 2012). In addition to out-of-body experiences, electrical stimulation at TPJ also induced vestibular, visual, and kinesthetic hallucinations (Blanke et al., 2002). Vestibular processing and out-of-body experience were linked at the phenomenal level in a different study on healthy individuals. Cheyne and Girard (2009) found that humans suffering from sleep paralysis (i.e., a sleep disorder that is associated with immobility after awakening from sleep) often experienced vestibular-motor hallucinations as well as out-of-body experiences. According to self-report these experiences occurred mostly in supine posture and began mostly with vestibular-motor hallucinations that were followed by out-of-body experiences.

Out-of-body experiences most frequently occur in supine posture when otolithic vestibular signals are altered with respect to the vertical body axis (Green, 1968), suggesting that otolithic vestibular processing is critical for these changes in bodily self-consciousness (Lopez et al., 2008). Together, these reviewed data suggest that altered vestibular processing at temporoparietal cortex is associated with disturbances in bodily self-consciousness during out-of-body experiences.

#### Experimental data

Similar changes in bodily self-consciousness can be studied in healthy humans using different body illusions, such as the body-swap illusion (Petkova and Ehrsson, 2008), the out-of-body illusion (Ehrsson, 2007) or the full-body illusion (Lenggenhager et al., 2007). During a classic version of the full-body illusion (Lenggenhager et al., 2007) a participant views (from a third-person viewpoint) a virtual body being stroked at the back, i.e., visual stroking, and simultaneously feels stroking at his or her physical body, i.e., tactile stroking. Importantly, the visual stroking of the virtual body and the tactile stroking at participant’s physical body are spatially separated. Synchronous visuotactile stroking typically increases self-identification with the virtual body and increases self-location in the direction of the virtual body, when compared with an asynchronous stroking control condition (comprehensive reviews and comparison to similar illusions in Blanke, 2012; Serino et al., 2013).

**Figure 2:**
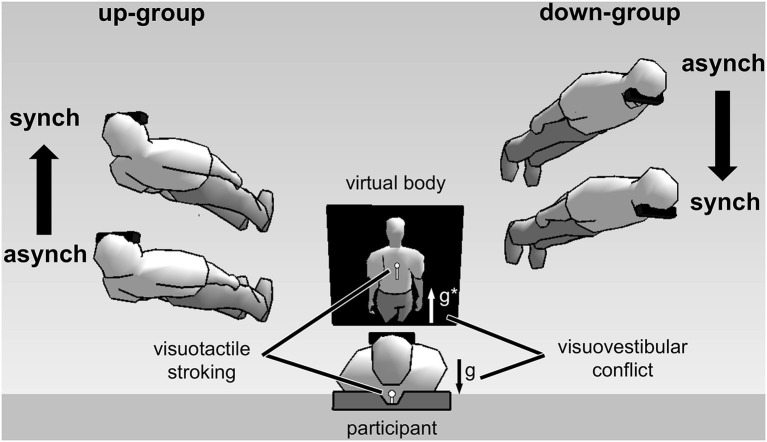
**Experimental setup and results of full-body illusion experiments using visuovestibular and visuotactile conflict (Ionta et al., 2011; Pfeiffer et al., 2013)**. (Image center shows) A participant in supine posture views a virtual body on a head-mounted display. Vestibular otolithic signals about gravity (g) are in opposite direction with respect to visual gravitational signals (g*)—thus in visuovestibular conflict. Results showed individual difference in first-person perspective experience. Virtual bodies at the left side of the figure represent subjective experiences made by up-group participants. These participants experienced an upward first-person perspective and showed congruent upward change in self-location during synchronous (synch) as compared to asynchronous (asynch) stroking condition. The opposite pattern was observed for down-group participants (shown at the right side of the figure).

Using such a full-body illusion setup we recently showed that the subjectively experienced direction of first-person perspective, together with self-location, was altered by directional conflict between otolithic vestibular and visual gravitational signals (Ionta et al., 2011; Pfeiffer et al., 2013). Figure 2 shows the experimental setup and results. Participants viewed a virtual body from an elevated visuospatial viewpoint, seeing visual gravity in downward direction, and simultaneously lay in supine body posture, receiving otolithic vestibular signals about their body being upward directed relative to gravity. Under these conditions individuals differed in terms of their experienced first-person perspective: up-group participants experienced an upward-directed first-person perspective and an upward-directed change in self-location during the full-body illusion. In contrast, down-group participants experienced a downward-directed first-person perspective and downward-directed change in self-location. Interestingly, individual differences in first-person perspective and self-location were reflected in changes in neural processing, as revealed by fMRI, in the bilateral TPJ, or more precisely in the posterior superior temporal gyrus (pSTG), a region close to the lesion overlap found in a group of patients with out-of-body experiences, i.e., angular gyrus (Ionta et al., 2011).

Pfeiffer et al. (2013) found at the behavioral level that individual differences in the subjective first-person perspective depended on individual differences in the way individuals weight visual and vestibular information, as assessed by subjective visual vertical judgments (Oltman, 1968). Participants oriented a visual line with respect their subjective vertical. A tilted frame around the line induced a small bias in subjective visual vertical judgments in some of the participants (visual independent group), while inducing larger subjective visual vertical biases in other subjects (visual dependent group). We found that assignment of participants to visual field dependent-independent groups, depending on their performance in the visual vertical judgment task, was predictive of their subjective first-person perspective during the full-body illusion. Specifically, participants from the visual independent group more likely experienced an up-looking first-person perspective during the full-body illusion, meaning that their subjective first-person perspective was congruent with vestibular signals. On the other hand, participants from the visual dependent group were more likely to experience a down-looking first-person perspective during the full-body illusion, meaning that their subjective first-person perspective was in line with visual signals.

Together, these studies support the hypothesis that the vestibular system contributes to whole-body spatial representation underlying bodily self-consciousness (Blanke et al., 2004; Blanke, 2012). One may wonder whether also body-part spatial representations depend on vestibular signals. Indeed, body-part representations are related to whole-body representations (Petkova et al., 2011; Ehrsson, 2012) and several studies observed vestibular effects on touch localization and shape perception of the hand (Lopez et al., 2010, 2012a,c; Ferre et al., 2011, 2013). However, these studies did not test whether vestibular stimulation also affected spatially integrated whole-body representations that underlie spatial aspects of bodily self-consciousness.

### Part one: conclusion and open questions

Multisensory conflicts, i.e., between vestibular otolithic and visual gravitational signals in healthy subjects, as well as vestibular hallucinations, i.e., due to functional interference at TPJ in neurological patients, have been associated with changes in bodily self-consciousness, most consistently regarding it’s spatial aspects: first-person perspective and self-location. Phenomenal experiences during these illusions included vestibular hallucinations, i.e., illusory reversals of the visuospatial first-person perspective with respect to gravity. Furthermore, ambiguous visual gravitational and vestibular otolithic signals induced changes of both first-person perspective and self-location. These observations suggest a critical role of vestibular cortical processing underlying spatial aspects of bodily self-consciousness.

Yet, very little is known about the functional and neural mechanisms underlying these effects. For instance, the vestibular peripheral system was never been directly stimulated during an out-of-body experience and a full-body illusion. It is thus not well studied how otolithic, semicircular, or both signals together affect spatial aspects of bodily self-consciousness. Furthermore, little is known about how vestibular processing contributes to a volumetric representation of the body, and how this spatial volume is related to representations of the external world. Finally, the vestibular system signals movement of the head and of the body. However, most studies on spatial aspects of bodily self-consciousness have used static body conditions. We think that these are important research questions for the future.

## Part two: vestibular contributions to bodily self-related cognitive and perceptual function

The second part of the review summarizes empirical research showing vestibular effects on mental spatial transformation, self-motion perception, and body representation. These cognitive processes involve spatial representations of the body, the external world, and the relationship between body and external world. We argue therefore that bodily self-related processes closely resemble spatial aspects of bodily self-consciousness, which require volumetric representation of the body with respect to the external world and spatial reference frames.

### Mental Spatial Transformation

Mental spatial representations are an important aspect of self-conscious experience. For example, the capacity to take the visual perspective of other humans is important for spatial cognition (Maguire et al., 1998), theory-of-mind (Baron-Cohen et al., 1985; Saxe and Kanwisher, 2003; Frith and Frith, 2006) and bodily self-consciousness (Newen and Vogeley, 2003).

Mental spatial representations have been extensively studied by mental imagery tasks involving objects, body parts, or entire bodies at different locations and orientations in external space (Shepard and Metzler, 1971). Mental imagery of these objects involves mental spatial transformation without participants actually moving their body or the perceived object. Performance in these tasks, i.e., reaction times and error rates, generally depend on the object rotation angle and the shortest path of rotation (Shepard and Metzler, 1971; Parsons, 1987a; Wexler et al., 1998). Mental imagery of body parts or entire bodies additionally depends on anatomical constraints of the physical body (Parsons, 1987b, 1994) and on participant’s body posture while performing the mental imagery task (Ionta and Blanke, 2009; Ionta et al., 2013).

A long tradition in cognitive neuroscience has studied egocentric imagery, which is self-centered mental spatial transformation of the own whole body or visuospatial perspective. In egocentric imagery tasks participants judge spatial attributes of objects in their environment from a location or perspective that differs from their actual location or perspective. For example, participants may judge whether a marker is at the left or right side of their imagined body location. Some researchers referred to egocentric imagery in the context of body-part imagery (Zacks and Michelon, 2005) which we argue does not necessarily draw on global representations of the whole body, but rather depends on body-part centered reference frames (Klatzky, 1997; Blanke, 2012). Therefore, we choose to refer to egocentric imagery for imagined own whole-body or perspective transformations. Egocentric imagery is typically compared to allocentric imagery, which is imagining transformations of objects in external space in order to judge their spatial attributes. Several studies have shown that egocentric vs. allocentric imagery depend on distinct functional neural activations (Mast et al., 1999; Wraga et al., 2005). For instance, egocentric, but not allocentric, imagery exhibits brain activity at the TPJ (Arzy et al., 2006)—the same brain region involved in spatial aspects of bodily self-consciousness, in out-of-body experience (Blanke et al., 2002, 2004; Blanke and Mohr, 2005) and in full-body illusions (Ionta et al., 2011).

While most previous research comparing egocentric with allocentric imagery focused on visual, motor, and proprioceptive contributions, more recent studies have shown very specific contributions of vestibular signals to egocentric mental spatial transformation. For instance, Grabherr et al. (2011) compared mental imagery in patients with vestibular loss (i.e., peripheral vestibular damage) with performance of healthy individuals (i.e., intact peripheral vestibular system). These authors found that bilateral vestibular impairment affected egocentric imagery when compared with unilateral loss or intact vestibular processing. Vestibular damage vs. intact vestibular processing did not affect allocentric imagery, thus highlighting the relevance of peripheral vestibular signals (intact or semi-intact) in egocentric imagery. Notably, egocentric imagery is known to rely on cortical activation of the TPJ (see above).

Likewise, highly specific effects of vestibular processing on egocentric imagery were found by Lenggenhager et al. (2008). Healthy participants received vestibular stimulation by left/right anodal GVS while viewing left/right rotated bodily or non-body object. Egocentric imagery was facilitated by side-congruent vestibular-visual stimulation, but only if participants viewed bodily objects. GVS had no effect on allocentric imagery and did not influence mental imagery of non-body objects. These results not only show vestibular modulation of egocentric imagery, but also vestibular processing specifically affecting body-related mental transformations for multisensory congruent directions. These results are congruent with clinical observations linking vestibular, visual, and kinesthetic processing at the TPJ and with changes of spatial aspects of bodily self-consciousness during out-of-body experience (Blanke et al., 2002).

While Grabherr et al. (2011) and Lenggenhager et al. (2008) studied the effects of vestibular damage and artificial stimulation on mental imagery, van Elk and Blanke (2014) used natural vestibular stimulation and found comparable results. Passive whole-body yaw rotations (activating the horizontal semicircular canals) facilitated egocentric body-related mental imagery if actual rotations and shortest paths of mental rotation were side-congruent. While general bilateral vestibular loss in the study by Grabherr et al. (2011), and GVS in the study by Lenggenhager et al. (2008), involved altered vestibular signals from both otoliths and semicircular canals, the study by van Elk and Blanke (2014) showed that selective stimulation of the semicircular canal signals affected egocentric mental imagery.

These data indicate that mental spatial transformation depends on vestibular signals. Vestibular processing enhances egocentric imagery when related to a visually seen bodily object. Vestibular signals from semicircular canals and otolith organs facilitate mental imagery in a spatial direction specific fashion.

Given that egocentric mental imagery draws on similar spatial representations and neural processing as spatial aspects of bodily self-consciousness, then it is likely that vestibular signals from semicircular canals contribute to spatial aspects of bodily self-consciousness and that they are processed at TPJ. To our knowledge, this hypothesis has not been studied directly. Instead, previous work on spatial aspects of bodily self-consciousness studied the effects of otolithic vestibular signals and representations of the static gravitational field.

Egocentric imagery recruits functional neural activation at the TPJ, suggesting that egocentric imagery engages similar representations, as do spatial aspects of bodily self-consciousness. Indeed, the strategy during egocentric imagery involves mental spatial displacement of one’s own body or perspective to a location in external space, whose analogous physical movements would activate otolithic and semicircular canals respectively. The effects found for rotational-direction specific contributions of vestibular signals to egocentric imagery suggest that cortical processing of semicircular canal signals may contribute to spatial aspects of bodily self-consciousness. Finally, vestibular signals facilitates egocentric imagery when viewing a human body shape, suggesting that egocentric imagery and spatial aspects of bodily self-consciousness are highly tuned to visual representations of the human body.

### Self-motion perception

Most everyday activities imply bodily movement in the environment. Planning and controlling these actions require accurate self-motion perception with respect to the environment and for this the brain must be able to monitor body movements based on multisensory signals. Furthermore, self-motion perception is important for balance, walking, and tracking the motion of objects under the influence of gravity. Research has shown that self-motion perception depends on integrating redundant sensory signals about body movement from vestibular, visual, proprioceptive, auditory and kinesthetic signals. Although vestibular signals alone indicate head posture and movement with respect to the environment, they are poor at sensing very slow movements (Kolev et al., 1996) and prolonged constant-velocity movements (Brandt et al., 1998). Similarly, the otoliths cannot distinguish between linear acceleration from head motion and constant gravitational acceleration (Einstein, 1907). Research on self-motion perception studied therefore multisensory integration mechanisms, i.e., most extensively visual-vestibular integration, in non-human primates (Andersen et al., 2000; Bremmer et al., 2002; Gu et al., 2007; Bremmer, 2011). These studies found that in the non-human primate brain the medial temporal region and dorsal MST region integrate optokinetic stimuli and vestibular signals about head rotation and heading direction. Another area integrating vestibular, visual, and somatosensory signals relevant for self-motion perception is VIP (Bremmer et al., 1999; Chen et al., 2013a). Neuroimaging in humans found comparable activation for visual-vestibular integration for self-motion in posterior parietal, parietooccipital, and medial temporal regions (Brandt et al., 1998; Kleinschmidt et al., 2002; Kovács et al., 2008; Becker-Bense et al., 2012).

While these studies showed that self-motion perception depends on an optimal comparison of dynamic multisensory stimuli, including vestibular signals about bodily movement, more recent studies have shown that also constant gravitational acceleration signals are important for self-motion perception. For instance, De Saedeleer et al. (2013) found that under normal terrestrial conditions (with constant gravitational acceleration acting in the downward direction), the velocity of perceived self-motion depends on the spatial direction of visual implied motion, and that self-motion velocity perception shows an asymmetric pattern for upward vs. downward, but not for leftward vs. rightward motion. Specifically, visual self-motion is experienced as slower when directed upwards (opposite to the downward direction of gravitational acceleration) than when directed downward (in the same direction as gravitational acceleration). In microgravity, when no otolithic vestibular signals are present, this upward-downward asymmetry is abolished. Interestingly, the transition between asymmetric to symmetric perceptual bias is delayed by several days when astronauts in microgravity are presented with tactile cues that mimic foot sole pressure, as if they were standing upright in a gravitational field. These results suggest that constant gravitational acceleration, but also multisensory cues, affect self-motion perception.

Neural correlates of self-motion perception as related to the gravitational field have been studied by Indovina et al. (2013). During fMRI, these authors presented visual self-motion cues in a virtual rollercoaster. For motion in the vertical, but not in the horizontal, direction the PIVC region was activated—a key region in the cortex receiving vestibular inputs. The activation depended on motion acceleration constant and showed strongest activation for direction-acceleration congruent motion at earth-gravity constant 9.81 m/s^2^.

Several studies from the same research group have previously shown that an internal model of gravity is recruited for visual motion perception. An internal model of gravity during these tasks recruited activation at of PIVC region, which was similarly activated by peripheral vestibular stimulation (McIntyre et al., 2001; Indovina et al., 2005). More recently, Maffei et al. (2010) found that visual object motion with a gravitational acceleration profile activated insula cortex and inferior parietal cortex. Both visually seen motion and unseen apparent motion cues similarly activated these regions. Activations were stronger when these signals were behaviorally relevant during an object interception task as compared to passive observation.

These recent studies in human subjects showed that self-motion perception is not only based on dynamic signals about body movement, but also on vestibular signal about the static gravitational field. Behavioral responses and functional neuroimaging suggest that the brain accounts for the effects of gravity on self- and environmental object motion by using an internal model of gravity that was found to overlap with cortical processing of vestibular signals in the PIVC region (Indovina et al., 2005, 2013)—a key region for vestibular input to the cortex (see Section Part Three: Vestibular Cortex and Spatial Aspects of Bodily Self-Consciousness of this review). Together, these findings suggest that vestibular signals about movement and position of the head are critical for self-motion perception, which draws on spatially representing one’s own-body movements with respect to the external environment.

Experiments on self-motion perception have extensively inquired about participants’ subjective experience of whether or not, and in which direction, they experienced to be moving. These are self-related perceptual judgments that are likely based on multisensory spatial representations of the bodily self (“I”) and the external world. Thus, self-motion perception likely draws on similar neural representations underlying the spatial aspects of bodily self-consciousness, i.e., self-location and first-person perspective. It is important for the brain to spatially update self-location and first-person perspective while the body is in motion, and to withhold from spatial update when there is motion in the environment. However, research on bodily self-consciousness has mostly studied static body conditions and thus to date the exact relationship between functional and neural representations of self-motion perception and spatial aspects of bodily self-consciousness is not well understood.

### Body Representation

Spatial aspects of bodily self-consciousness include a volumetric spatial representation of the body. Yet, no single sensory modality in isolation encodes such volumetric body representation. Instead, the brain integrates multisensory, body-related signals from the somatosensory, proprioceptive, visual, and, as it has been shown more recently, the vestibular system.

Longo and Haggard (2010) developed a task to assess perception of hand shape. They found that hand shape judgments were deformed in a manner partially resembling the cortical representation of the hand in primary somatosensory cortex. Using a similar task, Lopez et al. (2012c) studied the effect of vestibular stimulation by CVS on body representation and found that hand size judgments were generally enlarged by vestibular stimulation. A different study by Ferre et al. (2013) applied vestibular stimulation by GVS during a homologous task and found that finger representations were enlarged while hand dorsum was shrunk by vestibular stimulation. The specific differences between the results in these studies, i.e., enlargement or shrinkage of hand shape judgments, may reflect differences in the spatial directionality of the vestibular signals applied. Specifically, vestibular stimulation by CVS mostly activates the horizontal canals that encode yaw rotation, whereas GVS activates mostly the vertical canals (i.e., anterior and posterior canals) that encode roll and pitch rotation (Lopez et al., 2012b). Alternatively, these results may be based on additional factors to the stimulation technique utilized; for instance, sensory co-activation of thermal and nociceptive sensory signals. Despite differences between studies, both findings show that vestibular stimulation deforms hand shape representation. Thus, in addition to visual, somatosensory and proprioceptive signals (Serino and Haggard, 2010), the brain also integrates vestibular signals in order to determine the volumetric representation of the body.

Vestibular stimulation temporarily altered participant’s perception of the internal spatial configuration of the hand in the studies by Lopez et al. (2012c) and Ferre et al. (2013). These results differ from experienced changes of hand location during the rubber hand illusion (Botvinick and Cohen, 1998). Specifically, participants experience their own hand at a location different from their physical hand, but do not experience changes of hand shape. It seems that vestibular signals differently contribute to human position sense of implicit hand representations and overall hand location in external space. Two studies provide indirect support for this idea by showing that vestibular stimulation during the rubber hand illusion did not affect proprioceptive drift (Lopez et al., 2010, 2012a).

Generally, adult physical bodies undergo little change of shape over time, but vestibular stimulation immediately affected the internal representation of the hand shape. This suggests that highly plastic mechanisms underlie volumetric representations of the body. Such representations may be critical for spatial aspects of bodily self-consciousness, which can be manipulated rapidly during full-body illusions.

### Part two: conclusion

We reviewed data showing that vestibular signals from otolith organs and semicircular canals, as well as internal models of gravity, contribute to cognitive, sensorimotor, and perceptual functions. These self-related functions depend on vestibular processing at the TPJ, the intraparietal sulcus, the parietal-occipital and the medial temporal cortices. Because the TPJ also encodes spatial aspects of bodily self-consciousness, it is likely that vestibular processing at the TPJ is involved in both self-related processes and spatial aspects of bodily self-consciousness.

Vestibular signals are special sensory signals because the peripheral vestibular organs are fixed with respect to the head and therefore signal head movement relative to the external environment. Vestibular signals are thus likely to contribute in locating and updating location during movement of the body in the external world. However, vestibular signals alone are not sufficient, as they are signaling head position, but not the position of other body parts with respect to the external world. A multisensory integrated global representation of the whole body is necessary for bodily self-consciousness and thus vestibular signals need to be integrated with other spatially informative multisensory signals from the body. A full body representation can be achieved only by integrating multisensory body-related signals within a unique body-centered reference frame. Together vestibular world-related signals, when integrated with multisensory bodily signals, can provide a representation of the volumetric spatial body and its momentary position and orientation in space. Such representation of the whole body in space must be dynamically updated as the body and its parts continuously move. In this function, the vestibular signals are important to signal self-motion and thus to update spatial aspects of bodily self-consciousness with respect to the environment.

We think that for these functions, i.e., the spatial relationship between external world and a global full-body representation, and the update of the body-environment relationship in motion, vestibular processing in posterior brain regions is critical. In the final part of the present review we will present evidence supporting this view.

## Part three: vestibular cortex and spatial aspects of bodily self-consciousness

What are the neural correlates of vestibular processing contributing to bodily self-consciousness? Empirical data shows that in the right hemisphere posterior cortical regions process both vestibular signals and spatial aspects of bodily self-consciousness (Dieterich et al., 2003; Blanke and Mohr, 2005; Ionta et al., 2011). In the third part of this review we summarize the functional characteristics of three important posterior vestibular cortex regions, i.e., PIVC, MST, and VIP, and a region causally involved in bodily self-consciousness, i.e., TPJ, which together may encode self-location and first-person perspective.

### PIVC

It is commonly accepted that PIVC is a key region of vestibular input into the animal cortex (Grüsser et al., 1990a,b). This area also receives somatosensory and proprioceptive inputs (Lopez and Blanke, 2011). There is no consensus about the exact location and function of the PIVC in the human cortex. Different authors localized PIVC in the posterior insular and retroinsular cortex (Fasold et al., 2002; Indovina et al., 2005; Lopez et al., 2012b), in the parietal operculum (zu Eulenburg et al., 2012) and in different regions in the TPJ (Bense et al., 2001; Deutschländer et al., 2002; Lopez et al., 2012b). The available functional neuroimaging data in humans show that PIVC encodes vestibular signals from artificial stimulations by GVS and CVS (Fasold et al., 2002; Lopez et al., 2012b), proprioceptive signals from the neck (Fasold et al., 2008), and also visual signals (Brandt et al., 1998; Bense et al., 2001; Brandt et al., 2002; Deutschländer et al., 2002; Indovina et al., 2005, 2013). Although from non-human primate electrophysiology there is evidence for visual processing in PIVC (Grüsser et al., 1990a) there are also reports of no visual encoding in this region (Chen et al., 2010). Brandt et al. (1998) proposed that human PIVC and parietal occipital region encode visual and vestibular signals related to self-motion by a reciprocal visual vestibular inhibition mechanism. Specifically, these authors proposed that vestibular input activates PIVC and simultaneously deactivates parietooccipital region. Optokinetic stimulation, on the other hand, would activate parietooccipital region and simultaneously deactivate PIVC. Accordingly, the dynamic interaction between activation and inhibition from PIVC to parietooccipital region and *vice versa* would allow for determining self-motion. The PIVC projects to all other vestibular cortex regions, which is why some authors have discussed PIVC as the main vestibular input region to the human cortex (zu Eulenburg et al., 2012).

**Figure 3:**
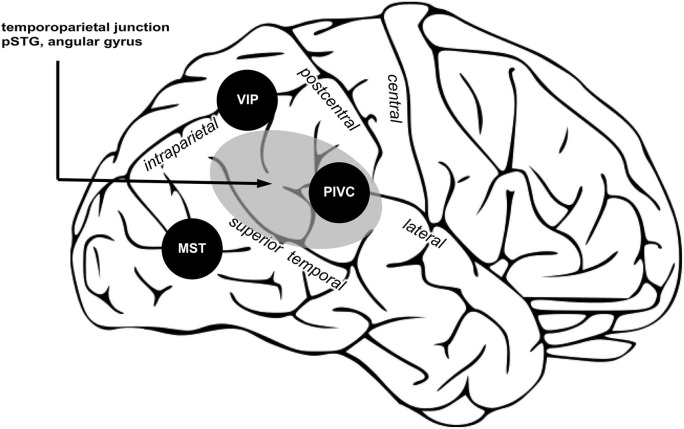
**Three posterior cortical regions processing vestibular signals are proposed important for bodily self-consciousness.** PIVC encodes vestibular signals about position and movement of the head; VIP, integrates multisensory signals and computes reference frames transformation to common body and world-centered spatial reference frames; MST integrate vestibular and visual signals necessary for self-motion perception. Area in gray shows the TPJ, an area causally involved in encoding spatial aspects of bodily self-consciousness. Within TPJ, the pSTG and angular gyrus are regions associated to changes in spatial aspects of bodily self-consciousness in out-of-body experience and full-body illusion, and also the vestibular cortex region PIVC is part of the TPJ. (Image is a derivative of work licensed under creative commons.)

What could be the role of PIVC in encoding the spatial aspects of bodily self-consciousness? Because PIVC can be considered a subregion of the TPJ (see Figure 3), on top of the evidence for PIVC as a major input area of vestibular signals into the cortex, in addition to PIVC’s strong connection to pSTG region, the PIVC seems to be critical in encoding vestibular signals contributing to self-location and first-person perspective. During experimentally induced changes in self-location and first-person perspective, vestibular otolithic signals play a critical role (Ionta et al., 2011) and these otolithic inputs as well as internal models of gravity have been reported to be encoded by PIVC and immediately neighboring regions (Indovina et al., 2005). It is thus likely that PIVC encodes body orientation and motion in the gravitational field and that these signals interact with neural processing regions at the TPJ coding for spatial aspects of bodily self-consciousness. Determining a clear functional and anatomical localization of PIVC in humans and its distinction from other neighboring regions involved in bodily self-consciousness will be an important goal for future research.

### MST

In non-human primates, the dorsal MST region is located in the extrastriate cortex. It processes visual optic flow stimuli, in addition to vestibular signals from body translation and rotation (Bremmer et al., 2002; Gu et al., 2007). Recent models proposed that MST neurons process the perceptual decision about self-motion by integrating visual and vestibular cues according to a Bayesian optimal integration model (Tanaka et al., 1986; Duffy and Wurtz, 1991; Gu et al., 2008; Fetsch et al., 2013; Chen et al., 2013a). While in primates next to MST also VIP neurons process optic flow, both regions are different in terms of their reference frame encoding such vestibular signals. While VIP encodes vestibular signals in body- and world-centered coordinates, MST encodes vestibular signals in eye-centered coordinates (Chen et al., 2013a,b). These data suggest that in primates, MST is a critical region of visuovestibular integration and self-motion perception. Due to morphological changes of the cortical structures between non-human primates and humans, the exact human homologue of MST (in terms of functional properties) is not precisely located in the human, however, functional neuroimaging studies have shown optic flow induced activity in the parietooccipital region (Brandt et al., 1998, 2002; Deutschländer et al., 2002). It is likely that the human homologue of MST is contributing to spatial aspects of bodily self-consciousness during self-motion by integrating visuovestibular signals. Therefore, vestibular processing in MST may play an important role in updating self-location and first-person while the body is in motion.

### VIP

VIP is a critical region for multisensory spatial coding. First of all, several findings both in humans and animals show that VIP processes visual, tactile, proprioceptive, and auditory stimuli (Duhamel et al., 1997, 1998; Bremmer et al., 2001; Avillac et al., 2005; Schlack et al., 2005; Sereno and Huang, 2006; Huang et al., 2012). A main function of VIP neurons is to integrate spatial information from different sensory modalities, which initially encode space in peripheral sensory system centered coordinates (e.g., visual stimuli in retinotopic coordinates; auditory stimuli in head coordinates; somatosensory stimuli in somatotopic coordinates) into common body-centered reference frames. Most neurons in area VIP respond selectively to visual stimuli presented close the animal’s body. Indeed, about half of VIP neurons respond best to visual stimuli within 30 cm of the body, and many neurons respond only within a few centimeters range (Colby et al., 1993). However, more distant space is also represented in VIP, since some neurons have visual receptive fields that are not confined in depth. In most neurons in VIP visual stimuli are encoded in body-part centered reference frames (typically centered at the head), some neurons are encoded in visual (retinal) reference frames, and some neurons have mixed reference frames (Avillac et al., 2005). Therefore, most VIP neurons preferentially represent the space near the body, in body-centered reference frames (Colby et al., 1993; Bremmer et al., 2002; Schlack et al., 2005). Although some neurons in VIP also encode visual-based representations of extrapersonal space, these extrapersonal space representations and the body-centered spatial representations are implemented in rather distinct neural populations within VIP (Colby et al., 1993), which supports the idea of distinct representations for near and far space, rather than a continuous representation from near to far space.

Interestingly, VIP also receives vestibular input. For instance, linear translations of the body, that are signaled by the otoliths, are encoded in VIP in body- or world-centered reference frames (Chen et al., 2013a). VIP may thus integrate vestibular with multisensory signals to compute spatial representations of the whole body—which are an important aspect of self-location (Blanke and Metzinger, 2009; Blanke, 2012; Metzinger, 2013). For all these reasons, computational models have proposed that VIP plays a critical role in coordinates transformation (Pouget et al., 2002; Avillac et al., 2005) and suggest that this region, together with other portions of the posterior parietal cortex plays a key role in remapping multisensory body-related signals into a common, whole-body centered, reference frames. Such computation is necessary to build a multisensory representation of the body in space, which is critical for spatial aspects of bodily self-consciousness.

### TPJ

The TPJ can be defined as a larger region including the pSTG, angular gyrus, supramarginal gyrus, and the parietal operculum (Figure 3, gray region). The TPJ receives somatosensory, visual, and vestibular inputs (zu Eulenburg et al., 2012; Bzdok et al., 2013). Note that PIVC is a subregion of the TPJ (Lopez and Blanke, 2011; Lopez et al., 2012b). The TPJ is important for multisensory signal coding (Downar et al., 2000), theory of mind (Saxe and Kanwisher, 2003), and bodily self-consciousness (Blanke, 2012). Several findings presented in the first and second part of this review show that damage or stimulation at the TPJ can induce changes in self-location and first-person perspective (Blanke et al., 2002, 2004; Ionta et al., 2011). In the same vein, changes in self-location and first-person perspective, induced in healthy subject by the full-body illusion, are encoded at the TPJ, and in particular in the pSTG region. Thus, the TPJ seems to be a critical region for encoding spatial aspects of bodily self-consciousness. We think that vestibular inputs from PIVC, MST, and VIP to the TPJ are critical for that function. In particular, TPJ might integrate inputs from VIP contributing to a global body representation, from MST to update body orientation and direction during movement, from PIVC for the orientation of the body in the gravitational field. When these vestibular inputs are absent or in conflict with other sensory signals, e.g., visual or somatosensory, the brain may generate an inaccurate spatial representation of the bodily self, inducing illusions in healthy participants or disorders of bodily self-consciousness in patients.

## Conclusion

The vestibular system processes head posture relative to constant gravitational acceleration and head motion in three-dimension space, thus providing important information related to the body with respect to the earth gravitational system, which is essential for coding the spatial orientation of the body in the external world. By reviewing recent data about bodily illusions, mental spatial representations, self-motion perception, and body representation, we argue that vestibular information is integrated with other sensory modalities to underlie bodily self-consciousness. Visual-vestibular interactions and internal models of gravity are processed at the TPJ, contributing to self-location and first-person perspective. We propose that this information depends on neural processing in the posterior cortical areas, which integrates and computes multisensory signals to build body representations in global whole-body centered reference frames and therefore contributes to stable representations of the bodily self. Integration of vestibular signals in PIVC, MST, and VIP, and further processing at the TPJ might be critical for the experience of the self as placed within a body, which occupies a specific location of space and faces the world from the first-person perspective. Vestibular processing may thus serve as a spatial reference for these spatial determinants of bodily self-consciousness.

## Conflict of interest statement

The authors declare that this research was conducted in the absence of any commercial or financial relationships that could be construed as a potential conflict of interest.
